# Selections and Abstracts

**Published:** 1897-11

**Authors:** 


### SELECTIONS AND ABSTRACTS.

---

### Treatment of Yellow Fever.

Dr. L. Sexton, of New Orleans, in the *American Medico-Surgical Bulletin*, says:

" As regards treatment, there is no set rule to follow. In fact, being a self-limited disease, there is nothing much to do but to treat it symptomatically. The least interference with its normal course is, perhaps, the most successful plan of procedure. If taken with a chill shortly after a hearty or indigestible meal, it is advisable to use a mild emetic of mustard or of salt and warm water. If constipated, a glycerin suppository or soap-and-water enema is indicated. Some prefer tasteless castor-oil, seidlitz powder, or citrate of magnesia. The initial treatment with many of our experienced doctors is 2½ grains each of calomel and soda.

As a rule, the majority of New Orleans doctors who have had experience with the fever, prefer the warm to the cold plan of treating it—*i. e.*, giving warm instead of cold drinks, until free perspiration is established. By flushing the alimentary canal and kidneys with pure cold water much good is done.

The first thing to do is to give a warm mustard foot-bath, in which the feet and legs up to the knees are kept in hot water for a half to one hour, if necessary. This usually results in free perspiration. Nausea is not likely to come on at this stage, but when it does, it is best treated with mustard over the stomach, or with teaspoonful doses of equal parts of cherry-laurel water and milk of magnesia. Crushed ice and iced champagne are recommended for nausea in persons accustomed to drinking. Bicarbonate of soda and peppermint-water suit some cases, especially where the stomach is acid.

In severe cases, where the temperature-range is high and pain is great, from 3- to 5-grain doses of phenacetin or 5-grain doses of acetanilid may be given within the first few hours, but not repeated.

Cold towels and water-bag to the back of the head, as well as the front, are very grateful to the patient. The arms may be sponged with cold water, but general sponging of the body with ice-water is to be deprecated. Alcohol and cool water should be used, instead. Kibbe died upon his own ice-cot, a memorable and lamentable example of trying to freeze out the fever. If there is a lagging on the part of the kidneys, 20 drops of turpentine in soft gelatin capsules with watermelon-seed tea is the treatment. Suppression of urine is to be watched for. Albuminous urine is common and to be expected, but when the urine begins to get scanty we must apply hot applications over the kidneys, give watermelon-seed tea, or some such simple diuretic as spirits of nitre and squills. The kidneys must be kept up to their duty, if possible, as anuria is almost certainly followed by death.

If the patient can successfully pass the sixth day, and is exceedingly careful about diet and exercise, he stands a fair chance of recovery. Muttering delirium, albuminous or suppressed urine are exceedingly unfavorable symptons, and may wind up a case fatally in a very short period of time.

Upon the theory that the infection exists in the bowels, and that intestinal disinfection is necessary, Dr. Sternberg recommends a solution of sodium bicarbonate and mercuric chloride in water internally four times a day.

To prevent auto-infection a brisk purge of sulphate or phosphate of soda is advisable, and naphthol and benzoate of soda have been used internally.

*Diet.*—Most of our doctors recommend a four days' fast. All of them only allow the most bland and liquid food. The theory of starving the patients and drenching them with water is the rule followed by many. The stomach is not in a condition to absorb food even if eaten. Since some is necessary to exist, those used with best effect are milk and lime or Seltzer water, barley water, flaxseed tea, weak tea, meat or chicken broth, soft-boiled eggs and egg albumen in cool water. As convalescence begins, the appetite is often voracious, but liquids and semi-solid food should be kept up to prevent relapses.

One reason children get well more quickly than grown people is the fact that they are not conscious of the prevailing excitement about the fever. Acting upon this theory, all discussions and conversation about death or spread of the epidemic should be avoided in the patient's room. The patient should not be allowed to take his own temperature, nor should the doctor take it oftener than four times a day, and the patient should not be told what it is if above 102° F. Not more than two persons should be permitted in the room at one time, and after the first two days it is well for the patient to be kept covered with a light spread or blanket. The bowels should be opened by a saline enema after the second day. Castor-oil and glycerin might be used as an enema if the salt fails to act.

Nausea may be controlled by 1- to 10-grain doses of calomel and $\frac{1}{8}$-grain doses of cocain floated into the stomach with cool water. Crushed ice should be supplied to cool the throat, provided the fever is high. If the heart is very weak and about to fail, 1-30 grain strychnine hypodermically, or $\frac{1}{2}$-grain doses of citrate of caffein.

In Havana, Cuba, several years ago, yellow fever patients were put in a room of cold air, or where temperature of room was reduced to the frost-line for the purpose of combating the fever. Its advocates claimed good results, but it has not been generally accepted. One of the methods of reducing high temperature here is the cold rectal enema of as much ice-water as can be conveniently held.

The surgical treatment of the fever may be shortly summed up into bleeding, either by cups, lancets or leeches. In the robust, where the pulse is bounding and temperature high, this helps them just as it might help in acute pneumonia. If collapse is imminent and hemorrhage great, the intravenous injection of normal saline solution at the temperature of the blood might avert a crisis.

---

### OPENING THE MASTOID CELLS.

Concluding "An Analysis of 114 Cases of Mastoid Involvement Complicating Acute Middle Ear Suppuration," Dr. J. E. Sheppard, of Brooklyn, says (*Medical News*, April 24) :

(1) For opening the mastoid the gouge or chisel has been invariably used, excepting only those cases in which the cortex was so much softened as to make the sharp curette more applicable. (2) For clearing out the mastoid, sharp curettes were used for the outer, blunt curettes for the deeper, portion. (3) *Grippe*, outside of the acute affections of the nose and nasopharynx due to cold, etc., has played the most important rôle in the etiology. (4) The symptoms are important in about the following order: (*a*) pain, either in the mastoid or in some part of the affected half of the head; (*b*) tenderness, either of the whole or of some part of the mastoid (oftenest of the apex); (*c*) drooping, or bulging, of the posterior superior canal wall into the lumen of the canal, especially if this is near the membrane; (*d*) posterior superior perforation of the membrane, especially if pouting or teat-like; (*e*) pulsating tinnitus, when it continues as a marked symptom after the time it should have ceased, were the case one of simple middle ear inflammation; (*f*) the external symptoms—redness and edema of the mastoid, and pushing outward of the auricle. (5) Abortive measures should be employed in a majority of cases for from two to five days, and will, as my cases show, be successful in curing a fair proportion of all cases. Having failed to give relief after the judicious use of abortive treatment, I believe the following may be justly given as the remainder of my conclusions: (6) We must time and again operate on the strength of one or two symptoms, and often with even these only slightly marked. (7) If a patient is seen before the external manifestations of mastoiditis have developed, we should never wait for their appearance, because by so doing we give the pus the same opportunity for breaking through the internal, as through the external mastoid cortex, the patient's life being meantime jeopardized to just that extent. (8) The operation, if performed with due care, is relatively free from danger. Danger to the patient arises, not from the operation, but from delay in performing it. (9) Finally, my cases make me believe that a pretty safe general rule would be, *when in doubt, operate.*

---

Patient: "Doctor, my breath is troubling me a great deal."
Doctor: "Oh, well, we'll soon stop that."—*Ex.*

### THE SURGICAL TREATMENT OF FIBROID TUMORS OF THE UTERUS.

Dr. Augustin H. Goelet, of New York, in a paper read by invitation before the Mississippi Valley Medical Association, at Louisville, enumerates as the surgical treatment of this class of pelvic tumors, curettage, division of the uterine arteries, myomectomy and hysterectomy. The conservative trend of the author's views is apparent. He thinks the use of electricity, in these conditions at least, should be limited to inoperable cases where the constitutional condition or cardiac disease prohibits, or where the patient refuses to consent to operation.

He believes the prejudice against operation is being rapidly removed, because the feasibility of conservative procedures has been demonstrated, and because the mortality of hysterectomy has been so greatly reduced. Attention is directed to the fact that an extensive myomectomy upon an uterus, the seat of numerous fibroids, is a successful possibility now, whereas only a few years back the uterus would have been sacrificed in such cases.

Curettage is designated a palliative measure against hemorrhage and endometritis, but it should constitute an auxiliary to all operations of a less radical nature than hysterectomy.

For permanent obliteration of the uterine arteries complete or almost complete atrophy of the tumor is claimed when it is appropriately limited and positive destruction of the continuity of the vessels is secured. Division as well as ligature of the vessels is insisted upon as essential to secure permanent arrest of the circulation. Simple ligature cannot be depended upon, because some broad ligament tissue is always included, and when the included tissue shrinks in consequence of the compression, the ligature loosens and the circulation in the vessels is frequently restored.

This operation should be limited to small interstitial growths and small subperitoneal tumors which spring from the uterine wall below the fundus. Those growing from the fundus or near the fundus are nourished chiefly by the ovarian and not the uterine arteries.

The removal of very large tumors of the submucous variety, which fill the uterine cavity, through the abdomen by incising the uterine wall and saving the uterus is regarded as preferable and more certain work than attempting to empty the uterus through the vagina.

The author does not think vaginal hysterectomy for uterine fibroids is a necessary or justifiable operation, since tumors which are sufficiently small to permit removal in this manner either need not be interfered with, or if they are causing symptoms, atrophy may be secured by dividing the uterine arteries or an abdominal myomectomy will save the uterus.

Abdominal hysterectomy should be reserved, he thinks, for cases where these other operations are not feasible, and especially where the size of the tumor makes it a source of great inconvenience and where degenerative changes have taken place.

---

### The Use of Opium in the Diarrheal Diseases of Children.

In no other condition is so much discrimination demanded in prescribing opium as in the diarrheal diseases of infants and young children. Much opposition to its use has been elicited during recent years, and many practitioners have discarded it entirely. Much of this opposition is, unquestionably, well founded. While its improper use may do great harm, when properly used it is an agent too potent for good to be wholly abandoned. We had occasion some time since to present in brief form its indications and contraindications in diarrhea as follows:

It is contraindicated—1, in the first stages of acute diarrhea, before the intestinal canal has been freed from decomposing matter; 2, when the passages are infrequent and of bad odor; 3, where there is a high temperature or cerebral symptoms are present; 4, when its use is followed by elevation of temperature or the passages become more offensive—symptoms which indicate toxic infection from putrefying intestinal contents.

It is indicated—1, when the passages are frequent, with pain; 2, when the passages are large and watery; 3, in dysenteric diarrhea, together with castor-oil or a saline; 4, in late stages, with small, frequent, nagging passages; 5, when the passages consist largely of undigested food, and the bowels act as soon as food is taken into the stomach.

The most important contraindications are admirably described by Dr. Miller. In the cases he reports, the dangerous symptoms were, no doubt, due to the absorption of toxic principles generated in the intestinal canal. The locking up of such putrefying matter by the use of opium violated every right principle of practice. Diarrhea would have been a conservative process, and frequent movements should have been encouraged. He clearly shows the reason for possible bad results in the following sentence: "Opium arrests the intestinal movements, and prevents the elimination of these toxic materials, and may give rise to grave symptoms of intoxication and sepsis."

The dose should be as small as possible, sufficient being given to relieve pain and check peristalsis. It should not be added to the ordinary diarrhea mixture, to be repeated at short intervals. It should be given alone, and at intervals sufficient to allow the effect of one dose to partly subside before another is given. This interval will be rarely less than four hours.—*Pediatrics.*

---

### TWO CLINICAL CASES UNDER PEPTO-MANGAN.

A year or more ago there came to our sanitarium a lady, past middle life, who had gradually became so thoroughly weak and anemic from chronic hepatic and digestive troubles that she could scarcely get about at all. After the correction of her functional troubles she did not gain strength, nor could I get anything to help assimilation and restore the lost balance until I placed her upon Pepto-Mangan, which she used for about a month, and left the institution greatly improved in every way.

Miss W., of New Kent Co., this State, applied to us for treatment for general debility and nervousness, following an attack of "Grippe," not the French *La Grippe*, but genuine American Grippe, which we have taken the liberty of coining to suit our ideas of this detestable trouble. This lady was completely used up, and could not undertake anything requiring effort or concentration. General health very bad, nervous system suffering intensely, digestion and assimilation, *nil.* Headaches, constipation, sleeplessness and fearfulness all added to her weakened condition, made her very miserable and unhappy. A few bottles of Pepto-Mangan started her speedily on the road to recovery, and she is now in very fair condition, and only taking a little strychnia as a placebo, and really not in need of anything.—*Dr. C. A. Bryce, Richmond, Va.*

---

### Homatropine in Refraction.

Jackson (*Journal of the American Medical Association*) gives an exhaustive article on the value of homatropine as a cycloplegic. When its use was first begun by him it was thought that it was less powerful than other mydriatics, and in many cases where there was any doubt stronger cycloplegics were used, but the results of the two examinations did not bear out the statements that homatropine has a weaker effect than atropine or similar drugs. Jackson believes that a great deal of the effect of a mydriatic can be varied by the mode of application, and that the original method of dripping a solution into the lower cul-de-sac is by no means sufficient to get the full effects of the drug. He applies the drug at the upper border of the cornea,the patient looking strongly downward. A solution of two to five per cent. is used, four to six installations being sufficient. The refraction should be determined within two hours of the last application. He does not believe that homatropine is as reliable as other mydriatics when used by the patient at home.

The writer believes that homatropine is a better drug than atropine for use in cases where complete rest of the accommodation is needed, because its action being much shorter the eye returns to its normal accommodative power without passing through a long period of ciliary muscular weakness. Of course the great advantage of homatropine is the brevity of its action. In the vast majority of cases the effects have completely passed off in less than three days, and in a large majority of cases the recovery is complete within forty-eight hours.

Toxic symptoms from homatropine are almost unknown. In Jackson's 2000 cases about one in 400 had some slight symptoms. Only one case showed marked symptoms, and these were not severe and passed off within three hours.

Homatropine is dangerous in cases where the tendency to glaucoma exists, just as all other mydriatics are, but it is less dangerous because its effects are of such short duration and its action can be easily neutralized by a solution of eserin.

Homatropine does not produce conjunctival irritation as other mydriatics do. It will cause a temporary hyperemia, but this generally disappears by the time the full effect of the drug has been obtained. There is usually no smarting nor any other evidences of inflammation.—*Medicine.*

---

### DEFECTIVE EYESIGHT IN CHILDREN.

Defective eyesight in children is a matter attracting much attention at the present time. The subject has been more or less forced upon the notice of the general public owing to its increase. The question of sight affects directly or indirectly every one. Through school and college course, in the choice of a profession or trade, to both sexes and in whatever sphere of life, vision is of paramount importance. That eyesight is degenerating is universally acknowledged, and as a matter of course the eyes of chidren share in this degeneration. It is one of the conditions inseparable from our advanced state of civilization, and that this is so is proved by the fact that the eyesight of those leading a primitive life or of those who have been only receiving the benefits of education and progress for a few generations has suffered no or little injury. The intellectual activity of a people may be very fairly gauged by its eyesight, the higher the brain development the worse the power of sibht and *vice versa*. A nation of students and bookworms as the Germans, show an enormous percentage of sufferers from myopia, while on the other hand races existing in a more arcadian state, despite their bad sanitary conditions, supply but a very small percentage of such cases. Statistics bearing upon this matter are, as they are prone to be always, somewhat variable and conflicting. Dr. William Carhart found, upon examination of 1,000 American school children, 48.5 per cent. astigmatic, 44 per cent. hypermetropic, and 3.5 per cent. myopic. Dr. Frank Allport gives the average of myopics attending the public schools in the United States at 30 per cent., while the examination undertaken about a year ago of 8,000 children attending elementary schools in London revealed the fact that out of this number but 39.5 per cent. possessed normal sight; slight and moderate degrees of hypermetropia were found to be the most common defects, and myopia was comparatively rare. The point of most interest in these examinations was the large number of children who possessed only sub-normal vision. From statistics, although not agreeing in all details, sufficient information may be gathered to emphasize the fact that in all civilized countries the number of children with defective eyesight is very large. The present race are not the only sufferers, but these defects will be handed down to future generations, with what ultimate disastrous results it is impossible to foretell. Many of the causes of, or aggravating aids to, defective vision in children are not far to seek: they are badly lighted, unsanitary rooms, very small print, uncomfortable seats, faulty position, too long hours of study; all these details should be looked to, and the children's condition during school hours be rendered as perfect from a health and comfort point of view as possible. The difficulty then occurs, how is defective vision to be detected, for the early correction of the refractive error, and especially of the astigmatic element, is of great preventive value. The plan proposed by the Germans that there should be district medical men who should yearly examine and report on the state of the scholars' vision, must be put aside as too expensive. Dr. Allport's method of training school principals and rendering them competent to detect and notify cases is worthy of consideration. There are countless numbers of little ones whose eyes are quite unfit for the close work they have to do, who are compelled to get through their daily routine the best way they can, and any plan that can be devised to remedy this evil will be welcomed.—*Editorial in Pediatrics.*

### INCREASE OF INSANITY AND CONSUMPTION AMONG THE NEGRO POPULATION OF THE SOUTH SINCE THE WAR.

Statistics gathered from the superintendents of southern hospitals for the insane show that both insanity and pulmonary consumption have increased disproportionately among the negroes of that section of our country since the close of the civil war. Thus, according to the United States census, there were in 1860 only 44 insane negroes in the State of Georgia; in 1870 there were 129; in 1880, 411, and in 1890, 910. In North Carolina there were in 1880 91 colored insane; in 1885, 144; in 1890, 244; in 1895, 307, and in 1896, 370. In Virginia, before 1865, there were about 60 insane negroes in the asylums of that State, and now there are over 1,000. In the Eastern Hospital for the Colored Insane, in North Carolina, consumption caused 14 per cent. of the total number of deaths in 1884, while in 1895 it produced 27 per cent. of all the deaths, and this in spite of a reduced general mortality rate. In the Mississippi lunatic asylum from 1892 to 1896, consumption caused 42 per cent. of the total number of deaths among negroes, or an increase of 22 per cent. over the death-rate from this disease among the white population outside of hospitals for the insane (it being of course well known that insanity predisposes to phthisis), if the latter is estimated at twenty per cent. In the Alabama Insane Hospital, during three years and nine months, beginning October 1, 1890, there occurred 295 deaths among 1,700 white and negro patients. Of the 179 deaths among the white patients, 28 per cent. were due to tuberculosis, and of the 116 deaths among the negroes, 42 per cent. were due to the same disease.

From this and other evidence which is presented, it is concluded that both of these diseases have disproportionately increased since the war, and that in all probability the cause which led to one also led to the other disease. The writer holds that the cause of phthisis resides in a disintegrating nervous system, and cites a number of concurrent authorities, as well as clinical and pathological data, to prove his position, and, among other conclusions, he draws the following: That both consumption and insanity are closely allied, both in personal and in family history, to idiocy, hysteria, epilepsy, asthma, and the other diseases of the brain and spinal cord; that they are both produced by syphilis, alcohol, overwork, business vicissitudes, domestic troubles, mental anxiety, grief, disappointment, and excesses of all sorts—in fact, by an agent or influence which vitiates the brain or nervous system, and that those who are confronted with a new and higher civilization and who are compelled to adjust themselves to these new relations are excessively liable to fall victims to insanity or pulmonary phthisis.

The condition of the negro is viewed from these premises. Civilization is regarded as an accumulation of forces, and the older the civilization the greater its momentum and the higher its plane, and when a lower civilization is precipitated in the midst of a higher, like in the case of the negro, it is the throwing together of two forces which differ in power and in rate of motion. The lower, in order to preserve itself, must make an effort to adjust itself to the course of changes of the higher movement, and the strain which is occasioned by this effort of adaptation, falls on and vitiates the brain and nervous system, and this in turn gives rise to insanity and phthisis. The vices of alcoholism and syphilis, which are readily acquired by these people, accelerate the advent of these diseases by destroying the integrity of the brain and nervous system.

Viewing the condition of the southern negro from these standpoints, it is perfectly obvious why insanity should necessarily develop, and on no other grounds can we explain why consumption should follow in the wake of insanity. Those who are able to realize all the factors which would be called into activity by the environmental condition of the negro after the war, could, at the time it was made, have foretold the inevitable results which are now but too plain to every one. It is, in part, a repetition of what happened, and now happens, to the aborigines of North America, Australia and New Zealand, who in their unequal warfare with modern civilization have been and being fast decimated and exterminated by pulmonary phthisis.—*Abstract of paper read by Dr. Thos. J. Mays, of Philadelphia, American Medical Association, June 2, 1897.)*

### HOLOCAINE.

Holocaine is the latest local anesthetic. The hydrochlorate of holocaine appears as white crystals, soluble in hot water, sparingly soluble in cold water. Gutmann has reported his experience with it (*Deutsch. Med. Woch.*):

He has used it in 30 cases in men, of which 13 had a foreign body in the eye, 2 were suffering from keratitis, 7 were ophthalmic operative cases, and in 8 cases the eyes were healthy. One minute after the instillation of from 3 to 5 drops of a 1-per-cent. solution there was anesthesia of the cornea. A passing burning sensation was felt. The foreign bodies were removed without inconvenience, and the galvano-cautery was used in a case of corneal ulcer and in a case of keratitis. Tattooing was also performed in two cases of leucoma. In one case it was possible to compare tenotomy of the eye-muscles under cocaine and under holocaine. Less pain was experienced in the latter instance. The duration of anesthesia under holocaine is from five to fifteen minutes. The cornea remains moist and shining. The ocular tension is not diminished. The pupil remains unaltered and accommodation is unaffected. In a patient aged 48, 3 drops of a 2-per-cent. solution of cocaine were instilled into one eye; anesthesia appeared in 2½ minutes, and lasted 3¼ minutes, the pupil dilated, and the tension diminished. With 3 drops of a 1-per-cent. solution of holocaine instilled into the other eye, anesthesia appeared after a slight feeling of burning in 1 minute, and lasted 9 minutes, the pupil was not dilated, and there was no alteration in the tension. The rapidity of anesthesia with holocaine is an advantage. The dilatation of the pupil under cocaine for the removal of foreign bodies is a disadvantage. The lessened pressure is a drawback in cataract-extraction, but useful in operation for glaucoma. The author says that holocaine should not be used subcutaneously, as intoxication-symptoms were rapidly produced by a small dose in a rabbit. For external use he recommends it as a substitute for cocaine.—*Am. Med. Surg. Bulletin.*

---

*She:* "I don't think these photographs do me justice."
*He:* "It's not justice you want; it's mercy."—*Town Topics.*

### GYNECOLOGICAL GLEANINGS.

All pelvic congestions are venous, and the term "chronic inflammation," so far as it applies to the organs in that cavity, is a misnomer, because the arterial vessels are not involved in that process.—*Emmet.*

Distressing pelvic pains incident to flexions and versions of the womb are greatly alleviated by vaginal suppositories, containing one grain of morphia and two grains of the extract of belladonna. —*Goodell.*

Vaginal injections of bromide of potassium I have found of real benefit in cases of so-called irritable uterus, diffuse pelvic pains and hysterical neuroses in various parts of the body. Injections containing them are best administered at bedtime. I have repeatedly seen a refreshing night's sleep follow the vaginal injection of one drachm of bromide of potash to a pint of water.—*Munde.*

—*Med. Summary.*

---

### DOES THE NEGRO HAVE LOCOMOTOR ATAXIA?

Dr. C. S. Briggs, of Nashville, Tenn., states (*Nashville Journal of Medicine and Surgery*, March, 1897) that although syphilis is exceedingly common among the negroes of that State, he has yet to see, after more than a decade's practice, a single case of locomotor ataxia in a negro.

---

### GARGLE FOR FOLLICULAR TONSILLITIS.

Beechwood creosote, 8 drops.
Tincture of myrrh, 2 ounces.
Glycerin, 2 ounces.
Water, 4 ounces.

—LEVY, in *Journal de Médecine de Paris.*

---

Little Elmer.—Pa, what is a coincidence?

Professor Broadhead.—The fact that the green cucumber is ready to begin its work just about the time that the green medical student graduates is a good example of coincidence, my son.—*Puck.*

### BOILS.

A very practical note—one that appeals to the every-day practitioner—is one found in a late article from the pen of Dr. L. Duncan Bulkley of New York, on the non-surgical treatment of ordinary furuncles. This particular note reads:

"The objects aimed at by the treatment are, (1) soothing and protecting an inflamed area; (2) exclusion of air; and (3) a slight antiseptic action. For this purpose a moderately thick layer of absorbent cotton is taken, several times the size of the inflamed surface: for a medium sized boil a piece one by two inches, with the fibers running the long way. Upon the center of the cotton a considerable mass of the following ointment is spread by means of a spatula, and this is then laid over the boil, and held in place by strips of adhesive plaster across the ends, but not passing over the boil. The ointment referred to is generally composed about as follows: ℞ Acidi carbolici gr. v.–x., ext. ergotæ fld. ʒ j.–ʒ ij., pulv. amyli ʒ ij., zinci oxidi ʒ ij., unguent. aquæ rosæ ℥ j. M. Ft. unguent. The relief often given by this dressing is very marked; the ointment soothes and protects the irritated surface, while the layers of cotton take up any outside friction. If comfortable, and unless disturbed, this dressing remains untouched twelve or more hours, when it is removed and a freshly spread piece immediately reapplied. If there has been any discharge the surface may be very gently cleansed with absorbent cotton, but I do not allow any squeezing. In many instances with proper internal and general treatment the boil aborts, and subsides without discharging; when this does not happen it ruptures spontaneously in a relatively short time, and I practically never find it necessary to incise it. This treatment I use in all stages of boils, keeping the ointment on until the boil is quite healed. If other boils form I direct it to be applied early and by this means they are frequently aborted.—*Clinical Review.*

---

### REJECTIONS FOR LIFE INSURANCE.

Dr. John Homans, medical director of the New England Mutual Life Insurance Company, Boston, Massachusetts, says: "The presence of the following diseases and conditions in any person applying for insurance will cause the rejection of the applicant unless the applicant presents himself for an examination by the medical director at the home office:

1. Albumen in the urine; asthma, except rose cold and hay fever; caries, or necrosis of bone; delirium tremens at any time; diarrhea, present or chronic; discharge from ear; enlarged glands; enlarged veins (varicose) above the knee; epilepsy; fistula, hernia, unless reducible and a suitable truss is worn; insanity; intermittent pulse; opium, chloral or cocaine habit; paralysis; prostate gland disease; pulse below fifty or over ninety, stricture, and sugar in the urine.

2. Light Weight.—Persons fifteen per cent. under weight, who have lost relatives from consumption, and all persons who are twenty-five per cent. under weight.

3. Heavy Weight.—Persons twenty-five per cent. over weight, who have lost relatives from apoplexy or heart disease, and all persons who are forty per cent. over weight.

4. Consumption in the Family.—1. Where more than three cases have occurred. 2. Where the applicant is under twenty, and one case has occurred. 3. Where under thirty, and one parent has had consumption. 4. Where under thirty-five, and any two members have shown the disease. 5. Where under fifty, and both parents have had the disease.

5. Repeated attacks of renal or hepatic colic and the passage of gall-stone within five years, or a renal or vesical calculus within three years.—*Atlantic Med. Weekly.*

---

ACCORDING to my observations it may be said that syphilis is much less severe to-day than it was thirty years ago. We no longer, not even in large syphilitic clinics, see so many cases of malignant, of severe, and of malignant precocious syphilis, nor do we meet with the more profound and grave tertiary by any means as frequently as we did a quarter of a century ago. This diminution in the severity of the disease is largely due to our improved methods of treatment, to better sanitary and nutritive conditions, and to the greater attention which is paid to cleanliness and antisepsis. But further than this, there undoubtedly exists to-day in the tissues of many individuals a greater resistance to syphilitic infection than was possessed years ago. In other words, in many people a moderate condition of immunity against syphilis exists, which is due to the changes in the tissues and perhaps in the blood induced by syphilis in their more or less remote ancestors.—*R. H. Taylor, M.D., in Medical News.*

